# FSTL1 and TLR4 interact with PEDV structural proteins to promote virus adsorption to host cells

**DOI:** 10.1128/jvi.01837-24

**Published:** 2024-12-13

**Authors:** Chunyun Liu, Ning Kong, Hailong Liu, Yu Zhang, Wenzhen Qin, Wenli Zhao, Xinyu Yang, Yahe Wang, Xinyu Cao, Tian Liu, Yuchang Liu, He Sun, Wu Tong, Hai Yu, Hao Zheng, Daoliang Lan, Shengsong Xie, Guangzhi Tong, Tongling Shan

**Affiliations:** 1 Shanghai Veterinary Research Institute, Chinese Academy of Agricultural Sciences 118161 , Shanghai, China; 2 College of Animal & Veterinary Sciences, Southwest Minzu University 66336 , Chengdu, China; 3 Jiangsu Co-Innovation Center for the Prevention and Control of Important Animal Infectious Disease and Zoonose, Yangzhou University 38043 , Yangzhou, China; 4 Key Laboratory of Agricultural Animal Genetics, Breeding and Reproduction of Ministry of Education & Key Lab of Swine Genetics and Breeding of Ministry of Agriculture and Rural Affairs, Huazhong Agricultural University 47895 , Wuhan, China; 5 Department of Preventive Dentistry, College of Stomatology, Shanghai Jiao Tong University School of Medicine Affiliated Ninth People's Hospital 56695 , Shanghai, China; St. Jude Children's Research Hospital, Memphis, Tennessee, USA

**Keywords:** FSTL1, TLR4, PEDV, structural proteins, virus adsorption

## Abstract

Infection with porcine epidemic diarrhea virus (PEDV) results in enormous economic damage to the global swine industry. PEDV starts its life cycle by binding to the receptors of host cells and adsorbing onto the cellular surfaces. However, it is still unknown how PEDV adsorbs onto the surface of host cells and the mechanism beneath the interplay of host cell transmembrane protein with PEDV proteins. FSTL1, which is a secreted glycoprotein, participates in diverse pathological and physiological processes, including immune modulation and cell proliferation and differentiation. The transmembrane protein, TLR4, serves as a pattern recognition receptor recognizing a broad spectrum of pathogens, which exerts a crucial effect on the host immune system. In this study, we identified that FSTL1 promoted PEDV infection. Further studies demonstrated the interactive relationship between FSTL1 and PEDV structural proteins (N and S2). In addition, we also confirmed that TLR4 interacted with FSTL1 and PEDV N, S1, and S2 proteins on the cell surface. Moreover, FSTL1 promoted the interaction of TLR4 and PEDV and induced viral adsorption to host cells. This study offers explicit evidence that FSTL1 and TLR4 act as mediators for host cell adsorption of PEDV by interacting with PEDV N/S proteins.

**IMPORTANCE:**

As a highly infectious porcine epidemic diarrhea virus (PEDV)-induced intestinal condition of swine, porcine epidemic diarrhea (PED) results in a 100% death rate among suckling piglets and poses a serious economic burden to global swine farming. Therefore, it is essential to investigate the mechanism of virus infection, replication, and proliferation. Virus begins its life cycle by binding to the receptor of host cells and adsorbing onto the cellular surfaces. However, it remains unclear how PEDV adsorbs onto the host cell surfaces. This study revealed that host protein FSTL1 interacted with the PEDV N and S2 proteins, while TLR4 interacted with the FSTL1 and PEDV proteins (N, S1, and S2). Moreover, we thoroughly and methodically demonstrated that FSTL1 was engaged in the PEDV internalization and attachment processes by promoting the recognition of PEDV N\S proteins by TLR4 and induced the viral adsorption to host cells.

## INTRODUCTION

As a member of *Alphacoronavirus* from the *Coronaviridae* family, the porcine epidemic diarrhea virus (PEDV) is the pathogenic factor of PED (1, 2). Initially identified over four decades ago, PEDV has spread widely in Asia, Europe, and the Americas (3). Its mutant strains have emerged, which are more virulent, pathogenic, and contagious, bringing enormous problems to the global swine industry (4). Due to infection, the intestinal epithelium is destroyed, and the villus shortens, causing watery diarrhea for around a week, as well as vomiting, anorexia, and fever. There is no age limit to the severity of symptoms, while mortality rates near 100% often occur in suckling piglets less than a week old (5). The interaction between receptors and coronaviruses exerts a vital role in their pathogenesis and cross-species transmission, as well as their cell and tissue tropism. Through their spike proteins, coronaviruses can bind to a broad spectrum of carbohydrate and proteinaceous cell surface molecules. Therefore, it is urgently needed to explore the receptors enabling host cell entry of PEDV.

PEDV begins its life cycle by binding to the receptor of host cells and adsorbing onto the cellular surfaces. The genome of this virus is either delivered immediately into cells through membrane fusion or formed into an endosome by the cells, allowing the cellular viral entry and viral genome delivery (6, 7). Spike (S) glycoprotein on the surface of virions is the chief determinant for tissue and host tropism, exerting a vital role in the host cell entry of the virus (8). In addition, the S protein is highly immunogenic and serves as the primary target of neutralizing antibodies (9). It binds to the cell receptors during viral entry and later serves as the mediator of viral membrane fusion. S protein encompasses an S1 subdomain implicated in the receptor binding and an S2 subdomain implicated in the membrane fusion. Virus replication and transcription are mediated by the nucleocapsid protein (N), which is highly conserved. Viral RNA genome and N protein constitute the core unit of PEDV (5). Moreover, the exploitation of aminopeptidase N protein (APN) by PEDV as a functional receptor has been discovered. Its binding domain can reside within the C-terminal half region of PEDV S1 (10). Nevertheless, various studies have indicated that for some cells, not expressing APN had no effect on their infectability by PEDV (11–14), suggesting that APN is not essential for the PEDV cell entry, and there may be other receptors in the entry of these cells by PEDV. This virus could promiscuously bind to various cell receptors. It has been suggested that a number of host membrane proteins can serve as critical entry cofactors, with the exception of an identified receptor for productive coronavirus infection (15–17). However, it is still unclear how PEDV adsorbs onto the surface of host cells and what the mechanism behind the interplay of the host cell transmembrane protein with PEDV proteins is. Therefore, investigating the interaction mechanism contributes to clarifying the etiological and immune-evasion strategies exploited by PEDV.

Our previous studies have reported that the PEDV proteins can make interactions with multiple host factors (18), including EGR1 (19), PABPC4 (20), TRIM21 (21), BST2 (22), and PTBP1 (23), to facilitate its invasion of the host. Our mass spectrometry analyses of the LLC-PK1 cell pulldown assay also exhibited a significant upregulation and interaction of host factor FSTL1 (follistatin-like protein 1). As a secreted glycoprotein, FSTL1 participates in various pathological and physiological processes that include immune modulation and cell proliferation and differentiation (24). FSTL1 makes a vital impact on the central nervous system (25). Moreover, many inflammatory pathologies are associated with increased FSTL1 expression, suggesting that it could be a beneficial biomarker for a range of inflammatory disorders (26, 27). Increased FSTL1 expression actively causes inflammatory mediators to be overexpressed, which in turn inhibits chondrocyte differentiation and stimulates the development of osteoarthritis (24, 28). Studies during the last few decades have suggested that FSTL1 is a novel protein associated with inflammatory diseases activating the production of proinflammatory cytokines and chemokines (29). Until the present, there are no clear reports of FSTL1 interacting with the virus. However, whether FSTL1 affects PEDV replication remains unexplored.

Toll-like receptor (TLR), which acts as the pattern recognition receptor (PRR) for recognizing a broad spectrum of pathogens, exerts a vital impact on the host immune system (30). When pathogen-associated molecular patterns (PAMPs) are recognized, TLRs are activated and serve as the anti-infection defense line of the host to trigger a signaling cascade, causing the discharge of different inflammatory cytokines (31). TLR4, as a transmembrane protein, can be activated by assorted PAMPs of fungal, bacterial, and viral origins (32, 33). In this study, we attempt to investigate whether FSTL1 and TLR4 are implicated in the host cell entry and infection by PEDV.

In this study, we indicate that host protein FSTL1 facilitated PEDV infection. Subsequently, we identified that FSTL1 interacted with PEDV N and S2 proteins. We also confirmed that TLR4 interacted with FSTL1 and PEDV proteins (N, S1, and S2) on the cell surface. Furthermore, we thoroughly and methodically demonstrated that FSTL1 is engaged in the PEDV internalization and attachment processes by promoting the recognition of PEDV N\S proteins by TLR4 and subsequently induced the viral adsorption to host cells.

## RESULTS

### PEDV infection enhanced the expression of FSTL1

**Fig 1:**
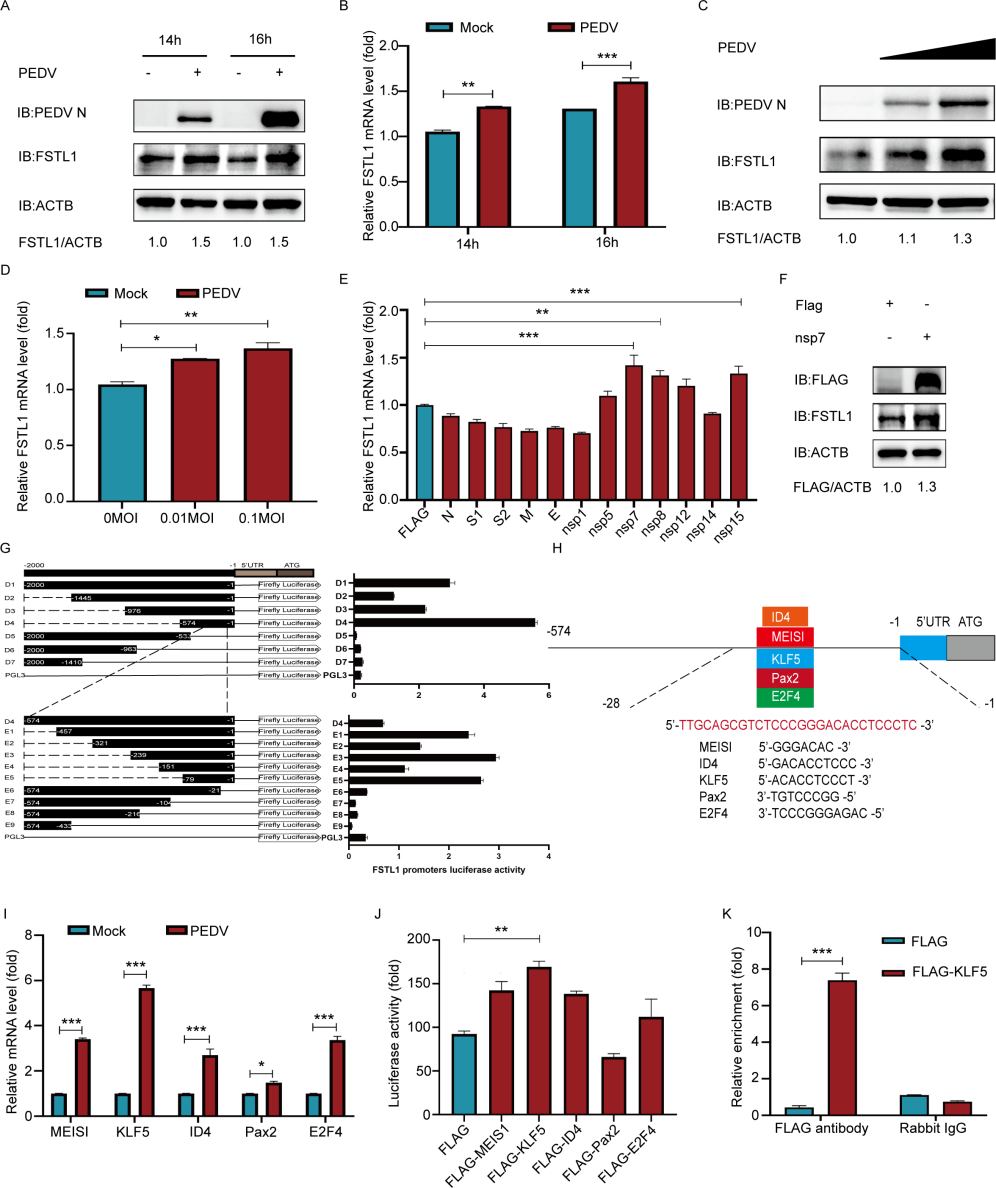
FSTL1 was upregulated by the PEDV nsp7 protein through transcription factor KLF5. (**A and B**) PEDV-infected or negatively infected LLC-PK1 cells (MOI = 1) were explored by western blot and qRT-PCR. (**C and D**) FSTL1 expression was determined through western blot and qRT-PCR in gradient PEDV-infected LLC-PK1 cells. (**E**) LLC-PK1 cells were transfected with PEDV structural and non-structural proteins, and then the mRNA expression levels of FSTL1 were identified by qRT-PCR. (**F**) Western blot was used to detect the expression levels of endogenous FSTL1 in nsp7-transfected LLC-PK1 cells. (**G**) HEK 293T cells transfected with pGL3-basic luciferase vector carrying truncated constructs (−2,000 to −1) of the FSTL1 promoter were explored for the luciferase activity. (**H**) The TFBS of the FSTL1 promoter was expected with JASPAR. (**I**) Relative mRNA levels of predicted genes investigated through qRT-PCR in LLC-PK1 cells infected with PEDV. (**J**) The luciferase activity was explored in MEIS1, KLF5, ID4, Pax2, and E2F4 over-expressing HEK 293T cells. (**K**) LLC-PK1 cells were transfected with FLAG-KLF5 plasmid and collected to perform a ChIP assay. Data are shown to be means ± SD of triplicate samples. *P < 0.05, **P < 0.01, and ***P < 0.001 (two-tailed Student’s *t*-test).

To explore the correlation of PEDV with FSTL1, LLC-PK1 cells were exposed to the PEDV challenge to examine the FSTL1 expression variations. The protein and mRNA levels of FSTL1 in the PEDV-challenged cells increased significantly (Fig. 1A and B), and the expression levels increased dose-dependently relative to the mock-challenged control (Fig. 1C and D). Next, plasmids capable of expressing PEDV structural and non-structural proteins were co-transfected into LLC-PK1 cells. The non-structural proteins nsp7, nsp8, and nsp15 significantly enhanced the FSTL1 at the mRNA level (Fig. 1E). Other proteins did not significantly enhance the transcription of FSTL1 mRNA. The endogenous FSTL1 protein expression was increased only in cells containing over-expressed PEDV nsp7 protein (Fig. 1F), suggesting that PEDV nsp7 protein was involved in upregulating the expression of FSTL1 during PEDV infection. In addition, we further explored the promoter region and transcriptional regulation of FSTL1. By amplification of the 2000 bp FSTL1 promoter gene and corresponding truncated sequences (D1–D7), the segments were inserted into pGL3-basic luciferase vector for direct luciferase activity assessment in HEK 293T cells. Based on the luciferase activities, D4 fragment was then truncated and inserted again (E1–E9) to determine the FSTL1 core promoter sequence range. Our results suggested that the core region of FSTL1 promoter spanned positions −79 to −1, which promoted the highest luciferase activity (Fig. 1G). Then, JASPAR (http://jaspar.genereg.net/) was employed to forecast the possible transcription factor-binding sites (TFBS) in the gene promoter (34). ID4-, MEISI-, KLF5-, PAX2-, and E2F4-binding sites were predicted in the core region of the FSTL1 promoter (Fig. 1H). Further qRT-PCR, chromatin immunoprecipitation (ChIP), and luciferase reporter assays on each predicted TFBS showed that only KLF5 among the above transcription factors was significantly upregulated and stimulated a significant higher-level FSTL1 luciferase activity in PEDV-infected cells, as well as directly bound to the FSTL1 promoter, which regulated its expression (Fig. 1I through K). The above results suggest the enhancement of FSTL1 expression by PEDV infection through transcription factor KLF5.

### Host protein FSTL1 promotes PEDV infection

**Fig 2:**
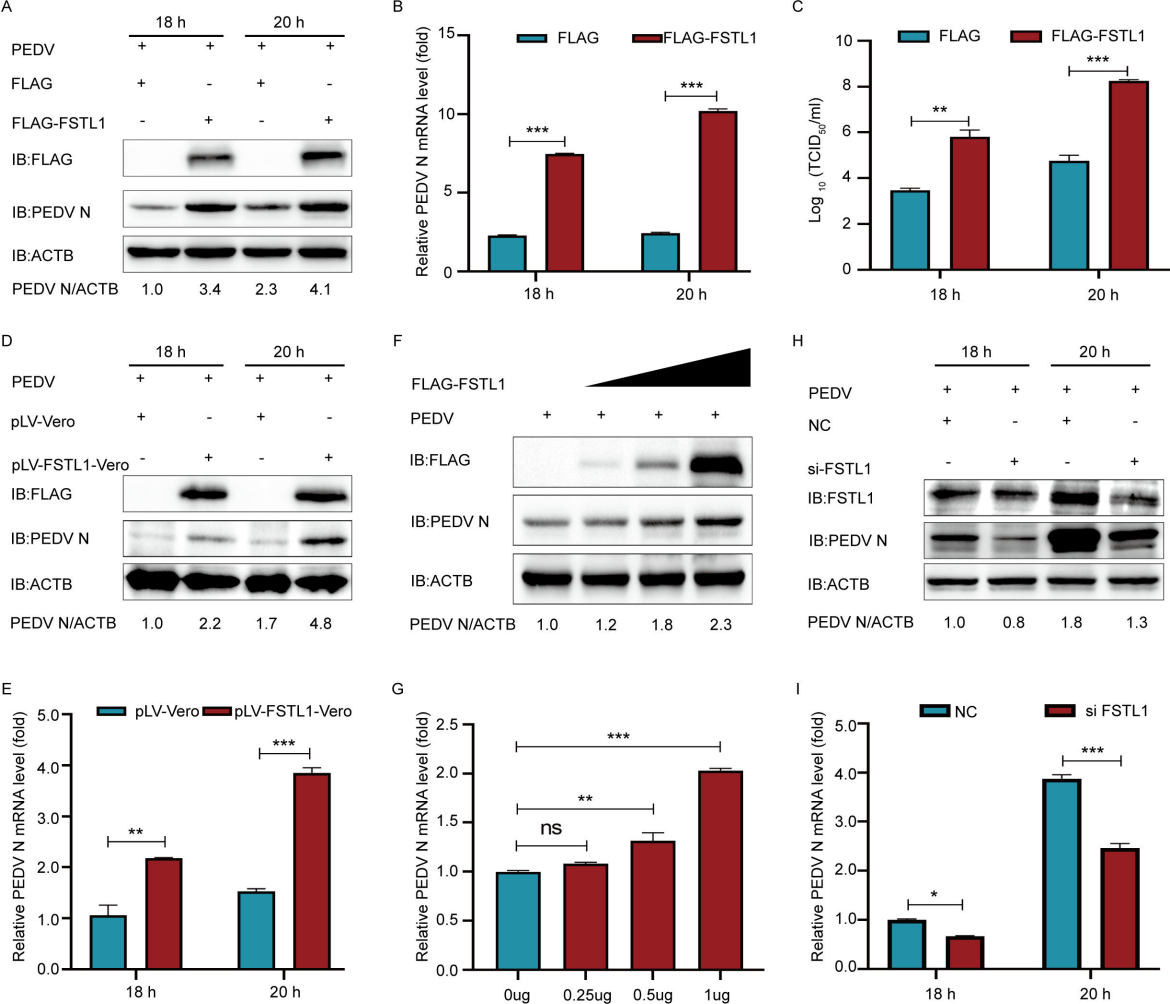
FSTL1 promotes PEDV replication. (**A–C**) LLC-PK1 cells were transfected with FLAG-FSTL1 plasmids and infected with PEDV at an MOI of 0.01. The PEDV titers were analyzed using western blot, qRT-PCR, and TCID_50_. (**D and E**) pLV-Vero and pLV-FSTL1-Vero cells were infected with PEDV at an MOI of 0.01. The PEDV titers were investigated with western blot and qRT-PCR. (**F and G**) LLC-PK1 cells were transfected with the gradient concentration of FSTL1 plasmids and infected with PEDV at an MOI of 0.01. The PEDV titers were explored using western blot and qRT-PCR. (**H and I**) LLC-PK1 cells were transfected with FSTL1 siRNA and infected with PEDV at the MOI of 0.01. The cell lysates were determined through western blot and qRT-PCR. Data are indicated as means ± SD of triplicate samples. *P < 0.05, **P < 0.01, and ***P < 0.001 (two-tailed Student’s *t*-test).

To investigate whether FSTL1 influences the replication of PEDV, FSTL1 was transfected into several cell lines transiently or stably to examine its impact on the virus. The Flag-FSTL1-transfected LLC-PK1 cells were further subjected to 0.01 MOI of PEDV 24 h post-transfection (hpt). The FSTL1-overexpressed cells showed significantly higher PEDV N protein and RNA copies than the control vector-transfected cells (Fig. 2A and B). A significantly enhanced viral titer was observed in the culture supernatant (Fig. 2C). To further clarify the biological implication of FSTL1 in the PEDV infection, the lentiviral system was employed to establish the FSTL1-overexpressing Vero cell line. Similarly, in FSTL1 over-expressed cell lines, the PEDV N protein along with mRNA increased significantly (Fig. 2D and E), indicating that FSTL1 facilitated PEDV replication. Moreover, the N protein and mRNA expressions increased with the increasing Flag-FSTL1 dose (Fig. 2F and G). Gene silencing was also performed to demonstrate the role of FSTL1. Small interference RNAs (siRNAs) were designed and used to knock down FSTL1 in LLC-PK1 cells to confirm its involvement in PEDV infection. As shown in Fig. 2H and I, the knockdown of FSTL1 was effective in inhibiting PEDV replication in LLC-PK1 cells. Collectively, these data suggest the positive regulation of PEDV proliferation by FSTL1.

### Host protein FSTL1 interacted with PEDV N and S2 proteins

**Fig 3:**
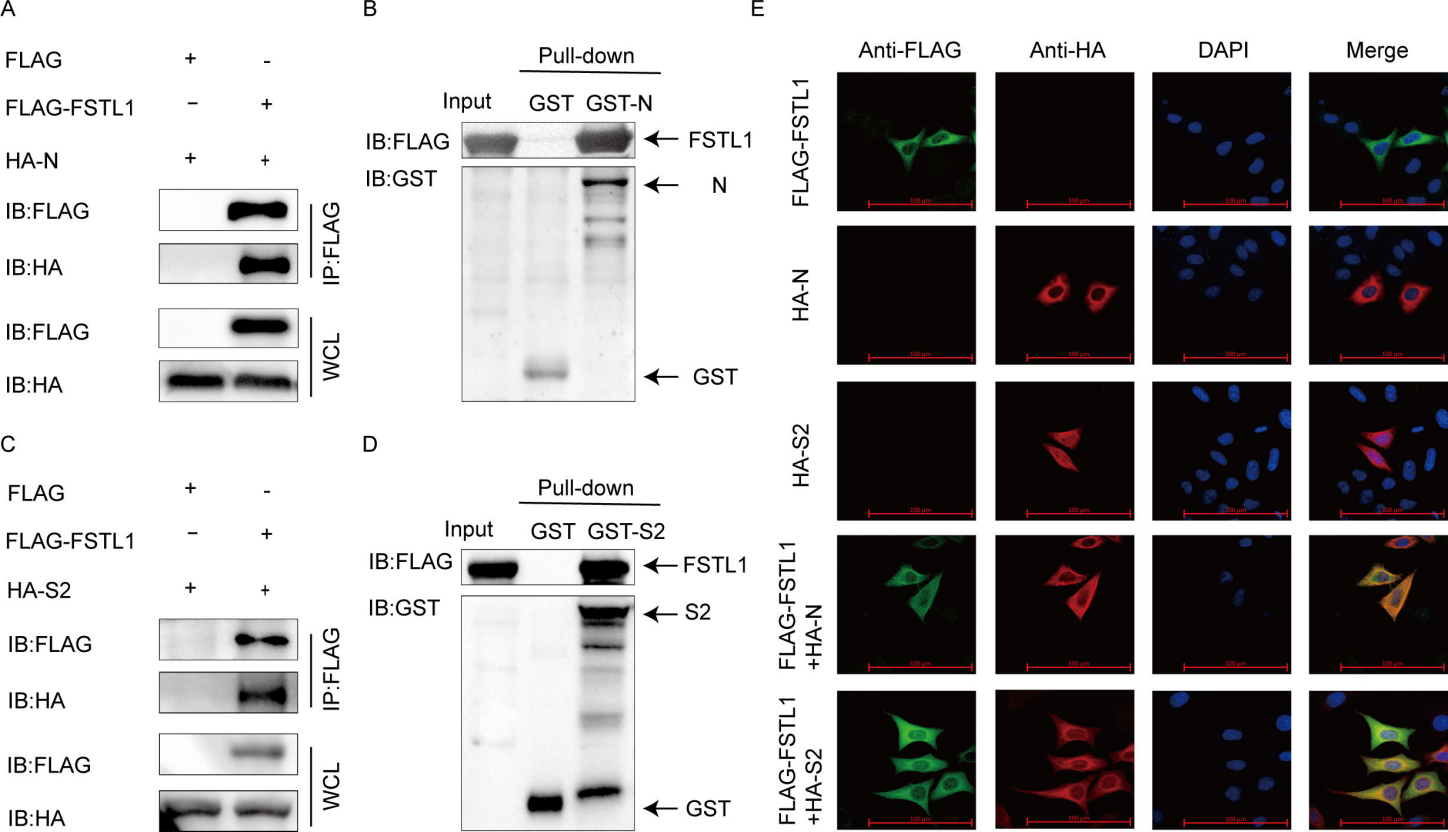
FSTL1 interacts with PEDV N and S2 proteins. (**A and C**) HEK 293T cells subject to transfection with the FLAG-FSTL1 and HA-N or HA-S2 plasmids. The cells were assayed by Co-IP with anti-Flag binding beads. Western blot was used to explore the precipitated proteins. (**B and D**) FSTL1, PEDV N, and PEDV S2 were cloned into pCold-TF or pCold-GST plasmids and denoted into BL21 (DE3) bacterial strain for affinity isolation of the GST pull-down assay. The eluted proteins were explored using western blot. (**E**) HeLa cells were transfected with FLAG-FSTL1 and HA-N or HA-S2 plasmids. The cells were labeled with specific antibodies. DAPI (4,6-diamidino-2-phenylindole) labeling for the cellular nuclei and fluorescent signals were monitored using a confocal immunofluorescent microscope (scale bars = 100 µm).

To reveal the interaction of FSTL1 with PEDV, the HEK 293T cells were co-transfected with plasmids, which could express FSTL1 and structural and non-structural proteins for co-IP analysis. Our results showed coimmunoprecipitation of the FSTL1 protein with the PEDV N protein (Fig. 3A). Furthermore, GST affinity-isolation assays were performed to demonstrate the interaction (Fig. 3B). Similarly, FSTL1 also interacted with the PEDV S2 protein (Fig. 3C and D). In addition, the interplays of FSTL1 with viral N and S2 proteins were further confirmed using confocal microscopy (Fig. 3E). The above results provide evidence for specific interaction of FSTL1 with PEDV N and S2 proteins.

### FSTL1 facilitates PEDV in the adsorption to host cells

**Fig 4:**
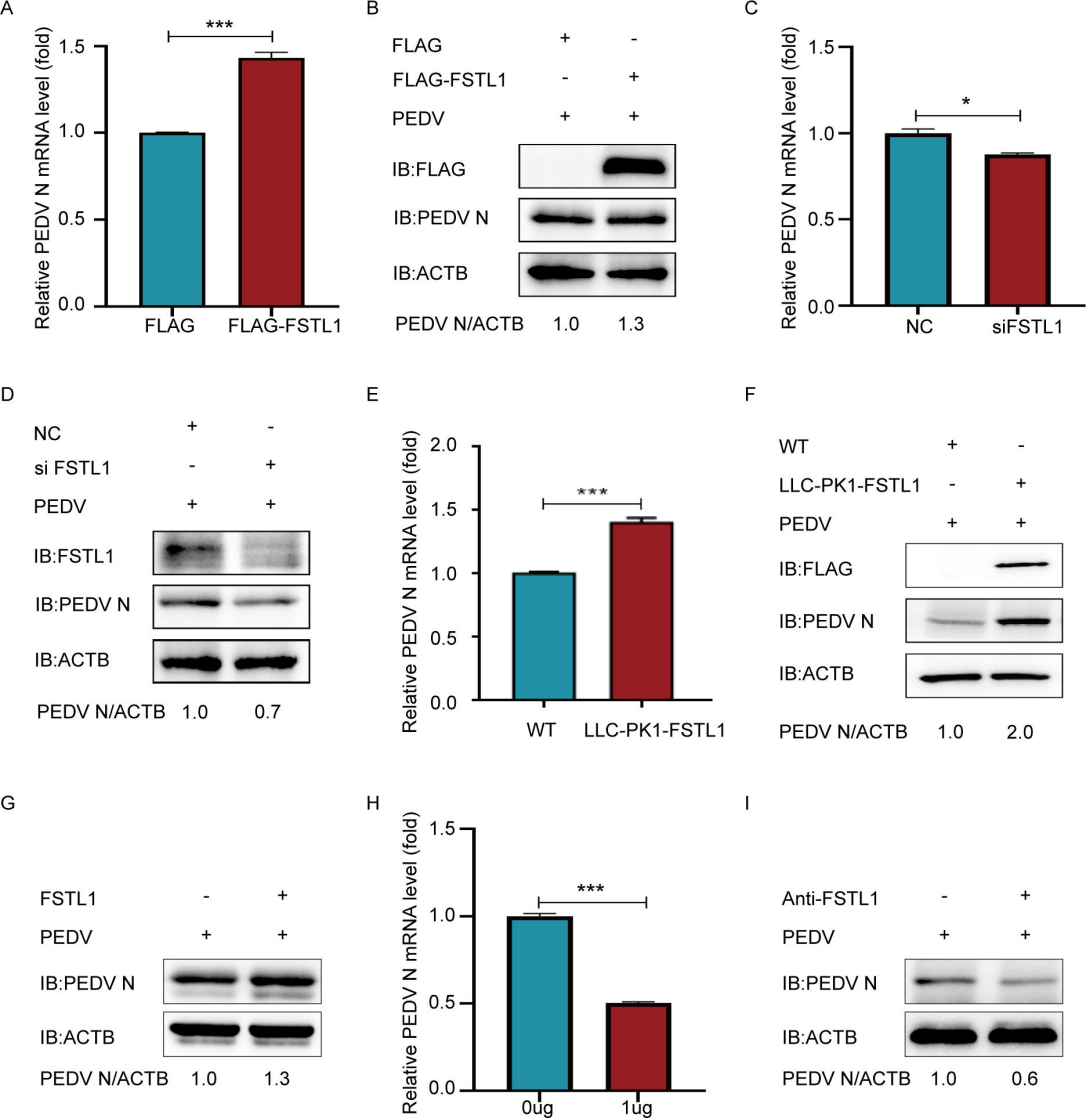
FSTL1 facilitates PEDV virion’s attachment to the cell membrane. (**A and B**) LLC-PK1 cells were transfected with FLAG-FSTL1 plasmids, infected with PEDV (MOI = 1) for 2 h at 4°C, and rinsed thrice with pre-cooled PBS to perform a virus adsorption assay. The PEDV titers were investigated using western blot and qRT-PCR. (**C and D**) LLC-PK1 cells were exposed to transfection with FSTL1 siRNA or siNC and infected with PEDV at an MOI of 1 at 4°C. The PEDV titer was tested using western blot and qRT-PCR. (**E and F**) The virus adsorption assay was carried out with FSTL-overexpressing cell lines, and the titers of PEDV were tested through qRT-PCR and western blot. (**G**) LLC-PK1 cells were pretreated with purified FSTL1 proteins at a concentration of 120 μg/mL and seeded with PEDV (MOI = 1) to conduct a virus adsorption assay. Western blotting was used to test the PEDV titers. (**H and I**) Detecting the attachment of viral particles based on qRT-PCR and western blotting after 1 μg of FSTL1 antibody occlusion in LLC-PK1 cells.

The processes of adsorption, cell entry, replication, and virion release comprise the life cycle of PEDV (35). To clarify whether PEDV infection is impacted by FSTL1 during adsorption phase, the FSTL1-overexpressing or -silenced plasmids were used to pre-treat LLC-PK1 cells for 24 h, which were subsequently exposed to PEDV (MOI = 1) for adsorption at 4°C. The findings of qRT-PCR and western blot used for infected cells indicated that overexpressing or silencing FSTL1 was relatively effective in the regulation of PEDV adsorption (Fig. 4A through D). In FSTL1 over-expressed Vero cell lines, the N protein levels of adsorbed virions were significantly increased in relative to the controls (Fig. 4E and F). This indicated that FSTL1 might benefit PEDV in the adsorption to host cells transiently or stably. Subsequently, we add prokaryotic-expressed FSTL1 protein to the viral culture medium. As displayed in Fig. 4G, FSTL1 significantly promoted the adsorption of virus to the cells. When the viral culture medium was pre-treated with the FSTL1 antibody, the protein and mRNA levels of adsorbed virions obviously decreased in host cells when compared with the control (Fig. 4H and I), indicating that blocking the function of FSTL1 decreases the adsorption of PEDV. All the above results suggest that FSTL1 facilitates PEDV adsorption to host cells.

### TLR4 interacts with FSTL1 and PEDV N and S proteins

**Fig 5:**
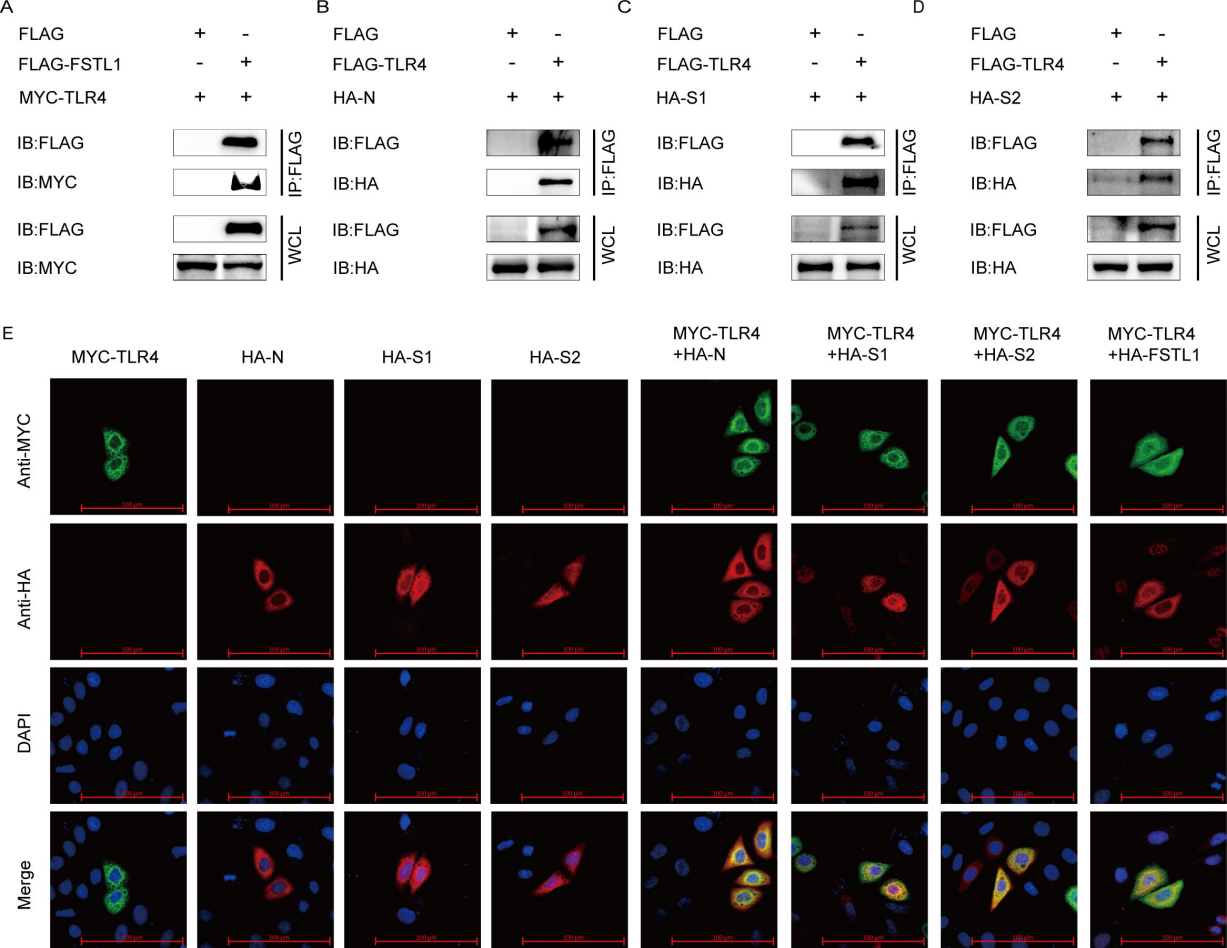
TLR4 interacts with PEDV N, S1, and S2 proteins. (**A**) HEK 293T cells were transfected with FLAG-FSTL1 and MYC-TLR4 plasmids. Co-IP assay was used for the analysis of the protein interaction. (**B–D**) HEK 293T cells were transfected with FLAG-TLR4 and HA-N, HA-S1, or HA-S2 plasmids. Co-IP assay was employed to explore the protein interaction. (**E**) HeLa cells were transfected with indicated plasmids. The cells were labeled with specific antibodies and monitored the fluorescent signals with a confocal immunofluorescent microscope (scale bars = 100 µm).

In view of the adsorption of PEDV onto the host cell membranes, it was speculated that there may be proteins present on the cell membrane aiding virus adsorption. Since FSTL1 is an extracellular protein, we explored which downstream protein on the surface of cellular membranes interplays with PEDV virions, as well as FSTL1 to assist PEDV entering host cells. TLR4, which is a PAMP receptor, has been demonstrated as the trigger of inflammation in several microbial infections, autoimmune diseases, and cancerous events (36, 37). Moreover, FSTL1 can accelerate aging of nucleus pulposus cells and degeneration of intervertebral discs through the TLR4/NF-κB axis (38). To determine whether TLR4 was involved in PEDV adsorption to the host cells, we examined the interaction between TLR4 and PEDV proteins, as well as FSTL1. Besides, the co-IP assay revealed coimmunoprecipitation of TLR4 with the FSTL1 and PEDV N, S1, and S2 proteins (Fig. 5A through D). We also observed colocalization of the PEDV N, S1, S2, and host FSTL1 proteins with TLR4 (Fig. 5E), demonstrating that the viral S and N could bind to TLR4 at the adsorption phase. These results indicate that TLR4 interacted with PEDV N and S proteins, which might form a functional complex during virus adsorption to host cells.

### FSTL1 promotes the interaction of TLR4 and PEDV N/S

**Fig 6:**
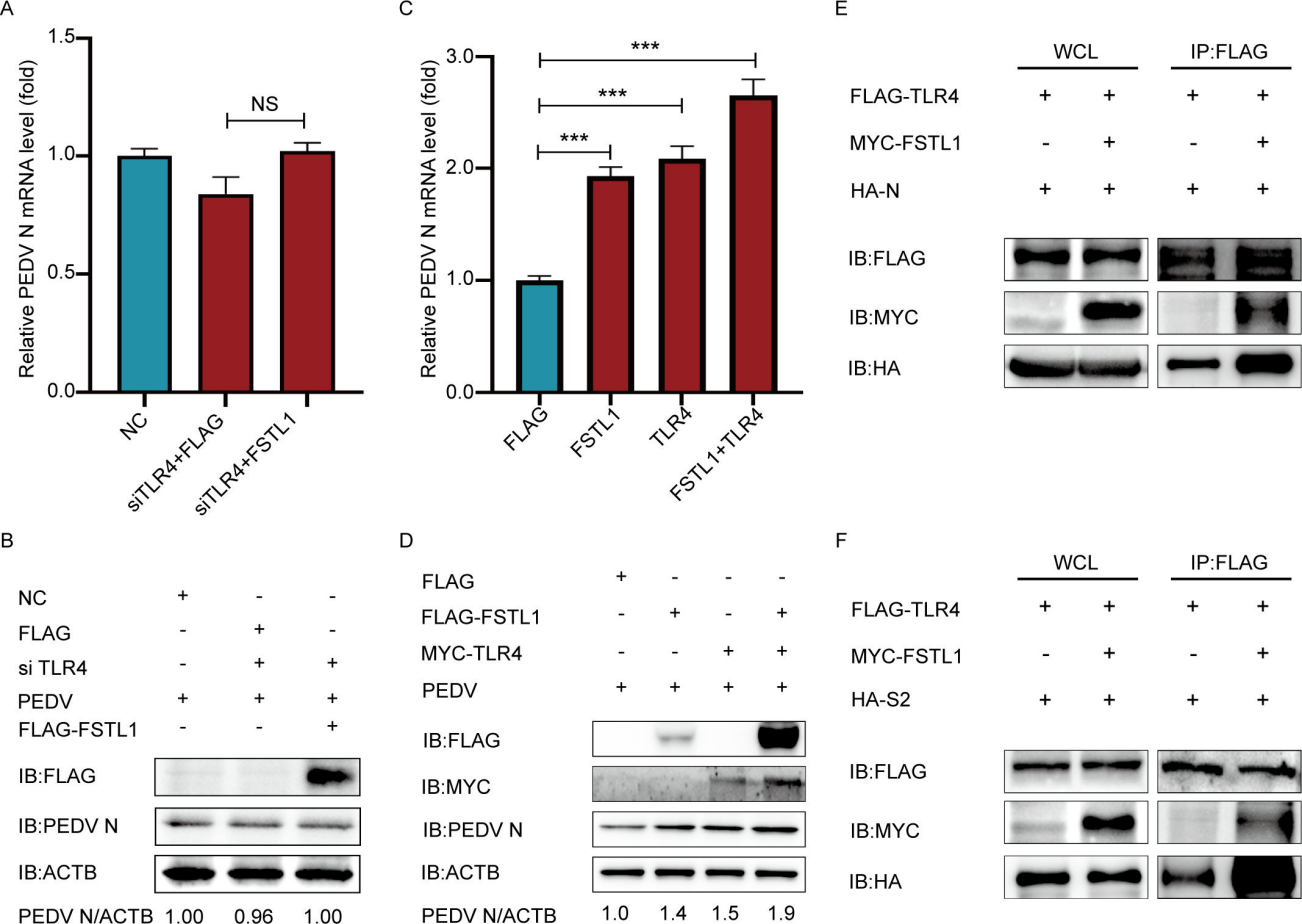
FSTL1 promotes the interaction of TLR4 and PEDV N/S. (**A and B**) LLC-PK1 cells were transfected with TLR4 siRNA and FLAG-FSTL1 plasmid, followed by infection with PEDV (MOI = 1) at 4°C for 2 h. The PEDV titers were determined by qRT-PCR and western blot. (**C and D**) LLC-PK1 cells were transfected with FSTL1 and TLR4 plasmids and infected with PEDV (MOI = 1) at 4°C for 2 h. The PEDV titers were detected using qRT-PCR and western blot. (**E and F**) HEK 293T cells were transfected with FLAG-TLR4, MYC-FSTL1, and HA-N or HA-S2 plasmids. Co-IP assay was used for exploring the protein interaction.

To further clarify the synergistic effect of FSTL1 and TLR4 during PEDV infection, TLR4 was silenced by siRNA. The qRT-PCR and western blot assays suggested that PEDV adsorption was not enhanced in FSTL1 over-expressed LLC-PK1 cells since the absence of TLR4 (Fig. 6A and B). Subsequently, we transfected LLC-PK1 cells with FSTL1 and TLR4 plasmids. Furthermore, we challenged the LLC-PK1 cells by 1 MOI of PEDV at 24 h post-transfection. The PEDV RNA copies and N protein in FSTL1-TLR4 overexpressed cells were the highest detected by western blot and qRT-PCR (Fig. 6C and D), indicating that FSTL1 may promote the PEDV adsorption by TLR4. To confirm this hypothesis, Co-IP assay was applied in TLR4-overpressed HEK 293T cells. As shown in Fig. 6E and F, the co-precipitated HA-tagged virus N or S2 protein could be more detected in MYC-FSTL1 transfected cells, suggesting that the interactions between TLR4 and PEDV N or S2 were enhanced by FSTL1. These results suggested that FSTL1 promoted the interaction of TLR4 and PEDV N/S proteins facilitating the virus to attach to the surface of the host cell.

## DISCUSSION

**Fig 7:**
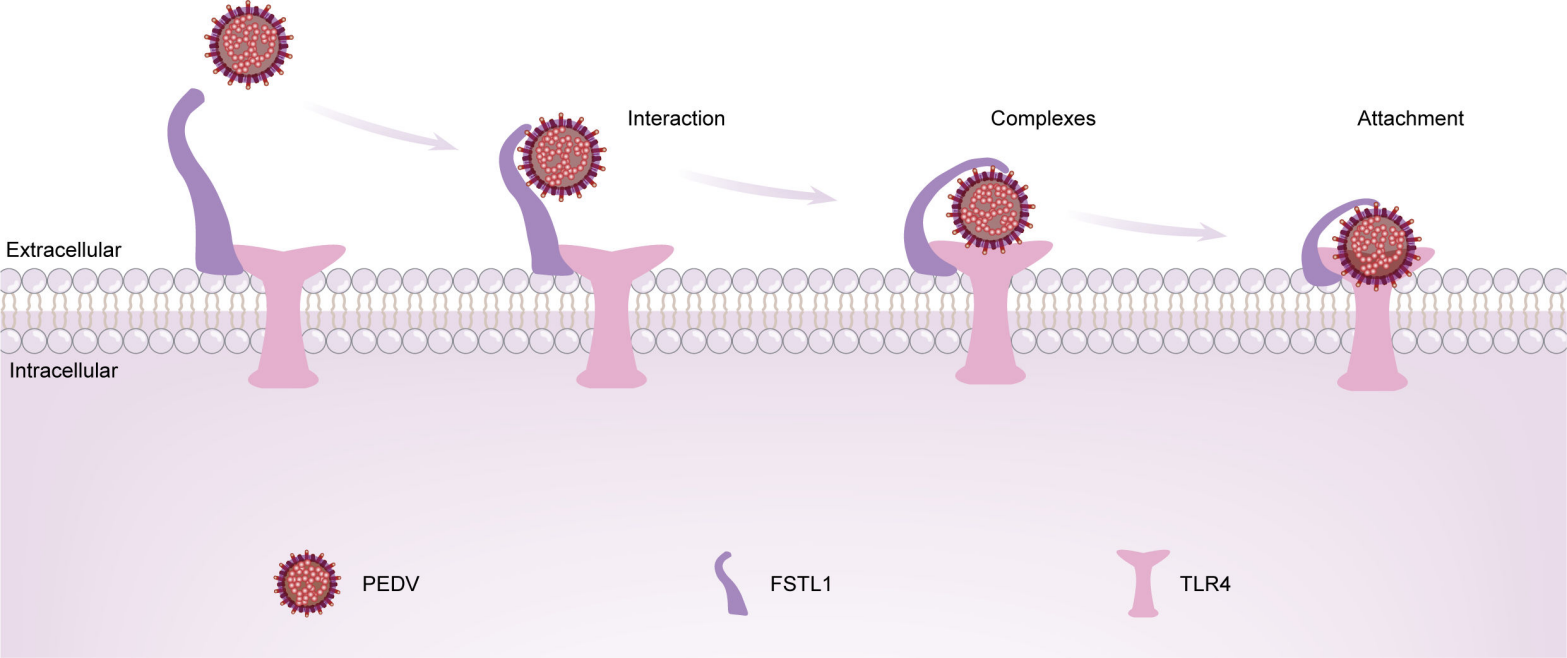
FSTL1 promotes attachment of PEDV by interacting with the TLR4 proteins. FSTL1 and TLR4 interact at the surface of the cell membrane. FSTL1 recognizes and interacts with PEDV virions that are free from the cell membrane. Then, FSTL1 promotes the interaction of PEDV virions and TLR4. Finally, FSTL1, TLR4, and PEDV virions form a complex that promotes the attachment of virions to the cell membrane.

The global pig industry has suffered tremendous losses due to PED, which is a widespread viral disease. Studies on the pathogenic mechanism of PEDV have been ongoing. Although host factors are gradually being identified as contributors to PEDV infection, few studies have investigated the interaction between PEDV structural and non-structural proteins and host membrane proteins, and less is known concerning the invasion process of PEDV (5). In this study, we first found that infection with PEDV enhanced the FSTL1 expression through the transcription factor KLF5. Since viruses hijack host proteins by using their own proteins and start multiple host biochemical events in the infection process to guarantee their efficient reproduction (39), this study investigated the relationship between FSTL1-related viral proteins and its related biochemical processes. Next, FSTL1 and TLR4 were found to be interacted with PEDV N and S proteins. Finally, we found that FSTL1 facilitated PEDV in the adsorption to host cells by promoting the recognition of PEDV proteins by TLR4 (Fig. 7).

The highly glycosylated trimeric S protein interacting with certain receptors on the surface of the host cell essentially enables the virus to fuse into the cell during the PEDV entry process (40, 41). Host receptor recognition necessitates the N-terminal S1 subdomain, whereas viral fusion necessitates the C-terminal S2 subdomain (8). This study first confirmed that extracellular protein FSTL1 interplayed with S protein and promoted PEDV infection, indicating a positive regulatory role of FSTL1 in PEDV replication. Further experiments showed a functional role of FSTL1, assisting PEDV in the adsorption to host cells both transiently and stably. Moreover, this study is the first to offer evidence for the extracellular action of FSTL1 with PEDV adsorption and infectivity, while its intracellular implication in PEDV replication and infectivity has scarcely been reported. As a soluble glycoprotein that is released into extracellular space, FSTL1 exerts a vital role in the development of many inflammatory illnesses and organ deterioration (42–44). FSTL1 possesses a multi-specific binding site that can bind to transforming growth factor beta (TGF-β) superfamily (45).

Next, we confirmed that the intermediary transmembrane protein, TLR4, which also interacted with cellular surface FSTL1 and S proteins, is essential in the PEDV adsorption to host cells. It has been recently indicated that FSTL1 elicits an innate immune response by binding to TLR4 and its coreceptor, CD14 (46). FSTL1 can stimulate NF-κB in a TLR4-dependent manner in HEK 293T cells, increasing the production of IL-6 and IL-8 (47). Furthermore, an increasing number of research has revealed a link between FSTL1, TLR4, and NF-κB (48). A similar phenomenon was detected in the PEDV N protein. Nevertheless, the effect of the N protein during the PEDV invasion process remains almost unknown. This also provides clues for finding the receptors of PEDV. Moreover, the functional domain of TLR4 and FSTL1 for PEDV attachment needs to be explored. Further investigation is required to unravel precisely how PEDV activates TLR4 and FSTL1.

TLR4 is involved in innate immunity and mediates inflammatory responses by recognizing pathogen. TLRs are transmembrane proteins with two distinct domains: a Toll-IL-1 receptor (TIR) domain in the cytosolic region responsible for intracellular signaling initiation and an external leucine-rich domain binding ligands (30). Viral glycoproteins are capable of TLR4 activation (49). For example, TLR4 has been reported as a receptor for Chikungunya virus envelope E2 glycoprotein and a regulator of virus-triggered pro-inflammatory reactions in host macrophages (50). The severe acute respiratory syndrome coronavirus 2 (SARS-CoV-2) S protein stimulates long-term cognitive impairment triggered by TLR4 in mice mimicking the symptoms of coronavirus disease 2019 (51). Glycoproteins of Ebola virus (EBOV), dengue virus (DENV), respiratory syncytial virus (RSV), etc., have also been shown to activate TLR4 (52–54). Membrane-associated viral glycoproteins, namely, EBOV glycoprotein (GP), RSV fusion (F) protein, and SARS-CoV-2 S protein, have been reported to facilitate the viral envelope fusion with the membranes of host cells (55). Non-structural protein 1 (NS1) of DENV indicates a glycoprotein secreted as a soluble hexameric complex by cells in the infection process, whose hydrophobic regions are exposed for the membrane contact (56). Based on our results, PEDV N/S, FSTL1, and TLR4 form a complex to promote adsorption of PEDV. When TLRs are activated, they recruit adaptor proteins, including MyD88, TIR-domain-containing adapter protein (TIRAP), TIRAP-inducing IFN-β (TRIF), and TRIF-associated adaptor molecule (TRAM), in addition to protein kinases, which are located in the cytoplasm of immune cells and include Tank-binding kinase 1, interleukin-1 receptor-activated kinases 1, 4, and inhibitor of NF-κB kinase (57). Cytokine production, proliferation, and survival are subsequently triggered by downstream protein activation. In addition, certain signals also cause increased adaptive immunity. However, we did not rule out TLR4 and FSTL1 functions in the cytoplasmic after the virus entered the cell, probably participating in a few intracellular protein–protein interactions mediating various processes like autophagy or proteasomal degradation. More in-depth research is essential to clarify whether and how TLR4 and FSTL1 exert roles in regulating intracellular replication of PDEV.

### Conclusion

To conclude, our findings demonstrate, for the first time, that the secreted glycoprotein FSTL1 and membrane protein TLR4 interact with PEDV S and N proteins during PEDV infection. Furthermore, we identified the host proteins FSTL1 and TLR4 as new targets that regulate attachment and contribute to the internalization of PEDV through FSTL1 and TLR4. FSTL1 is a binding partner specific to PEDV proteins, which mediates viral adsorption to host cells by promoting the interplay of TLR4 with viral N/S proteins and subsequently triggers viral adsorption to host cells. This novel finding can expand our knowledge about PEDV infection, and the clarification of FSTL1’s and TLR4’s implication in PEDV adsorption and internalization provides an innovative foundation for the development of potential therapeutic drugs.

## MATERIALS AND METHODS

### Cell lines, antibodies, and virus

Vero E6 and HEK 293T cells were cultured in Dulbecco’s Modified Eagle Medium (DMEM) (Gibco, C11995500BT) containing 10% fetal bovine serum (FBS; Yeasen, 36101ES60). LLC-PK1 cells, which were provided by Dr. Rui Luo (Huazhong Agricultural University, Wuhan, China), were cultured within MEM (Invitrogen, 11095080). All these cells were cultivated in an atmosphere of 5% CO_2_ at 37°C. Anti-GST-tag (10000-0-AP), anti-FSTL1 (20182-1-AP), and anti-ACTB/β-actin (66009-1-lg) antibodies, along with horseradish peroxidase (HRP)-conjugated anti-mouse (SA00001-1) and anti-rabbit (SA00001-2) IgG antibodies used, were all Proteintech products.

JS-2013, a variant strain of PEDV used in this study, was isolated and preserved in our laboratory [63]. During viral infection, Vero cells were cultured to above 90% adherence in microplates, washed in PBS (Gibco, C20012500BT), and then challenged by 0.01 or 1 MOI of PEDV and treated with 4 µg/mL trypsin (Invitrogen, 15050065). After 60 min, the cells were washed with culture within serum-free DMEM for multiple times at 37°C and subsequently harvested.

### Quantitative real-time PCR

Total RNA was extracted following the protocols of the QIAamp Viral RNA Mini Kit (Qiagen, 52906) or the RNeasy Mini Kit (Qiagen, 74104), while cDNA was prepared through the PrimeScript RT Reagent Kit (Takara, RRO47A). For the qRT-PCR assessment, SYBR Premix Ex Taq (Vazyme Biotech, q711-03) was used. All primer sequences utilized in qPCR were presented in Table 1. ACTB served as internal references.

**TABLE 1:** Primers used for qPCR

Purpose	Name	Sequence (5′−3′)
qPCR primers	PEDV N forward	GAGGGTGTTTTCTGGGTTG
	PEDV N reverse	CGTGAAGTAGGAGGTGTGTTAG
	GAPDH forward	ATGGATGACGATATTGCTGCGCTC
	GAPDH reverse	TTCTCACGGTTGGCTTTGG
	FSTL1 forward	TACCTATCCAGACCAGGAGAACAA
	FSTL1 reverse	CGGTTACAGTCCACCTCAGTCTC
	KLF5 forward	CACCTCCATCCTATGCTGC
	KLF5 reverse	GCACTTGTAGGGCTTCTCG

### Western blot

After 5 min of cell incubation on ice by a protease inhibitor cocktail (Bimake, B14001)-supplemented RIPA buffer (Thermo Fisher Scientific, 89901), the lysates were collected for 10 min of denaturation within 5× SDS-PAGE loading buffer. Next, proteins were isolated through SDS-PAGE and subsequently shifted onto blotting membranes (GE Healthcare, 10600001). Membranes were blocked by PBS, which contained 0.2% Tween 20 (Sigma-Aldrich, P1379) and 5% skim milk powder (BD, 232100), followed by further incubation using primary antibodies under room temperature and HRP-conjugated secondary antibodies. Finally, proteins were measured with an ECL chemiluminescent substrate (Share-bio, SB-WB012).

### ChIP assay

PK-15 cells were seeded onto six-well microplates transfected with Flag or coding plasmids and, 24 h later, gathered for ChIP assay via the SimpleChIP Enzymatic Chromatin IP Kit (Cell Signaling Technology, 9003). Immunoprecipitation of chromatin fragments was performed by applying anti-Flag antibody-bound protein G magnetic beads (Cell Signaling Technology, 9006).

### Co-immunoprecipitation assay

After 24 h of transfection with plasmids, cells were lysed in protease inhibitor cocktail-supplemented NP40 buffer (Life Technologies, FNN0021). Lysates were collected for centrifugation and incubation by adopting anti-Flag-antibody-bound Dynabeads Protein G (Life Technologies, 10004D), then washed in 0.02% PBST and resuspended within a pH 2.8 glycine buffer (50 mM). Finally, the eluted proteins were immunoblotted by specific antibodies.

### Prokaryotic expression and purification of FSTL1 protein

Full-length sequences of FSTL1 were inserted in pCold TF plasmid and expressed in BL21-competent cells (Vazyme Biotech, C504-03). The prokaryotic FSTL1 protein was expressed in BL21 (DE3) induced by 0.1 mM of isopropyl-β-D-thiogalactopyranoside (IPTG; Takara, 9030) at 16°C for 20 h. After centrifugation and ultrasonication, FSTL1 protein was purified using Ni-NTA His·Bind Resin (Novagen, Madison, WI) according to the manufacturer’s instructions. The purified samples were analyzed for protein concentration using the BCA Protein Assay Kit (Thermo, 23227).

### GST affinity-isolation assay

Initially, full-length *S1*, *S2*, *N*, *FSTL1*, and *TLR4* gene sequences of PEDV were inserted in pCold TF or pCold GST plasmid. Afterwards, BL21-competent cells were employed to intracellularly express these genes. Afterwards, interactions between proteins were assessed following the protocols of the GST Pull-Down Kit (Thermo, 21516).

### Confocal immunofluorescence assay

Transfected cells were immobilized in 4% paraformaldehyde (Sigma-Aldrich, P6148), permeabilized with 0.1% Triton X-100 (Sigma-Aldrich, T9284), blocked in 5% BSA for 60 min, and subsequently incubated for another 60 min with primary antibody, followed by PBS washes for three times. Subsequently, the cells were incubated in dark for another 60 min with fluorescently conjugated secondary antibody based on a previous approach (22). After 5 min of nuclei staining with DAPI, fluorescence images were monitored by laser scanning confocal immunofluorescence microscopy (Carl Zeiss, Germany).

### Luciferase reporter assay

HEK 293T cells grown on 24-well microplates were transfected with target-encoding plasmids by using Lipofectamine 3000. At 24 hpt, cells were harvested for the luciferase activity determination based on the Dual-Glo Luciferase Assay System (Vazyme Biotech, DL101), in which the reference luciferase was *Renilla*.

### Statistical analysis

Intergroup comparison was made by two-sided Student’s *t*-test with the application of Prism 5 (GraphPad Software, USA). Significant differences were indicated by *P < 0.05, **P < 0.01, and ***P < 0.001, while non-significant differences were represented by ns. Data presented are means from three independent assays.

## Data Availability

All data are contained within the article.
